# Sporadic Pemphigus Foliaceus in a 3-Year-Old Vietnamese Girl: A Case Report and Literature Review

**DOI:** 10.1155/2024/6748340

**Published:** 2024-02-16

**Authors:** Trinh Thi Diem Nguyen, Trinh Ngoc To Chau, Phuong Thi Doan Vo, Hao Trong Nguyen

**Affiliations:** ^1^Ho Chi Minh City Hospital of Dermato-Venereology, Ho Chi Minh City, Vietnam; ^2^Pham Ngoc Thach University of Medicine, Ho Chi Minh City, Vietnam

## Abstract

Pemphigus foliaceus is an uncommon autoimmune intraepidermal blistering disease characterized by immunoglobulin (Ig) G autoantibodies that attack desmoglein-1 in the epidermis. There are two predominant forms of pemphigus foliaceus, sporadic and endemic. Sporadic pemphigus foliaceus is known to be more prevalent in middle-aged and elderly people and to be extremely rare in children. Less than 40 nonendemic pediatric pemphigus foliaceus cases have been documented in the literature. This report documents a case of sporadic pemphigus foliaceus in a 3-year-old Vietnamese girl who presented with generalized scaling and crusted erosions over the body.

## 1. Introduction

Pemphigus belongs to a heterogeneous group of immunobullous diseases where pathogenic autoantibodies attack the cell surface of keratinocytes, resulting in the disruption of cell-cell adhesion in the epidermis. Based on the levels of intraepidermal detachment and the types of antigens under attack, pemphigus is divided into pemphigus foliaceus (PF) or pemphigus vulgaris (PV). PF, characterized by more superficial blisters and lack of mucosal involvement, is milder than PV. The peak age of onset for both types of pemphigus is 40–60 years, yet it may also occur in younger patients, including infants. Childhood pemphigus remains rare, with an apparent preponderance of PV [1]. Due to the paucity of pediatric PF cases, misdiagnosis and diagnostic delays are common that may lead to serious complications [2, 3]. Moreover, data on treatment, long-term follow-up, and overall prognosis are still limited. Herein, we report a unique case of pediatric PF and review the currently available literature in an effort to elucidate the characteristics, clinical course, and treatment options for this condition.

Pemphigus erythematosus, with characteristics overlapping PF and lupus erythematosus attributes, is considered a separate condition rather than a variant of PF, and is thereby not included in this review [4].

## 2. Case Report

**Figure 1:**
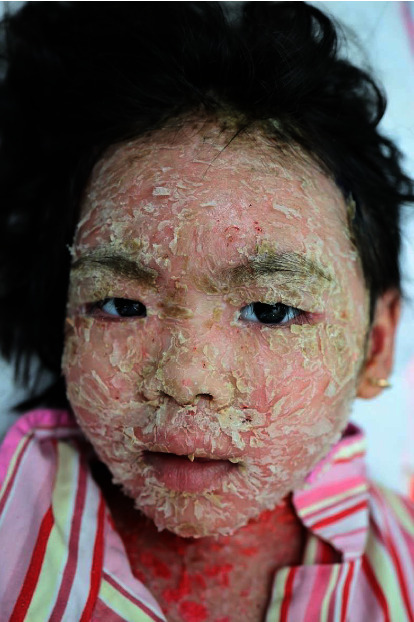
Large flaky scales in the background of diffuse erythema on the face of a pemphigus foliaceus patient.

**Figure 2:**
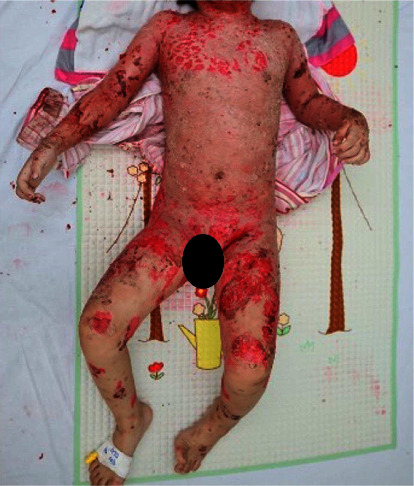
Diffuse scaly and crusted lesions on the trunk and extremities of a pemphigus foliaceus patient.

A 42-month-old Vietnamese girl was transferred to our hospital with extensive scaling and reddening of the skin. The skin lesions had erupted 8 weeks prior, initially appearing as scaly and crusted erythematous macules on her face. The patient was not taking any medications before these skin lesions developed, and her medical history was unremarkable. She was initially misdiagnosed with infected eczema and treated with various antibiotics for 6 weeks without improvement. Subsequently, flaccid blisters developed on the trunk and extremities, which promptly ruptured into diffuse crusted erosions. The patient was admitted to a pediatric hospital for severe skin infection and sepsis and was treated for 2 weeks before being transferred to our hospital. On examination, the facial skin was covered with large flaky scales in the background of diffuse erythema, and its tightness caused mild ectropion (Figure 1). Generalized scaling and crusted erythematous patches were interspersed with moist, eroded areas on the trunk and extremities (Figure 2). Skin lesions caused severe pain, leading to limited mobility. Nikolsky's sign was positive. No evidence of mucosal or nail involvement was found.

**Figure 3:**
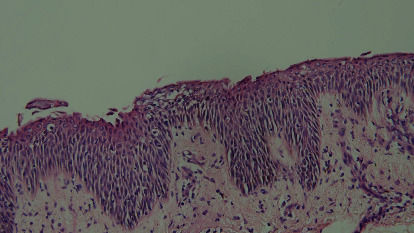
Skin biopsy of a pemphigus foliaceus patient with H&E staining shows a cleft within the subcorneal layer and superficial acantholysis. H&E, hematoxylin and eosin.

**Figure 4:**
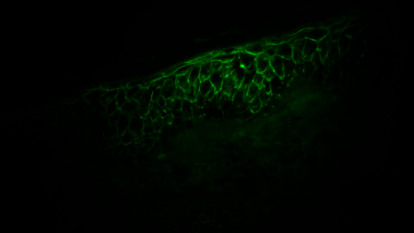
Positive DIF findings demonstrate C3 and IgG deposition in the upper epidermis of a pemphigus foliaceus patient. DIF, direct immunofluorescence; Ig, immunoglobulin.

Microscopic examination of the scaly erythematous lesions showed multiple focal splits at the level of the subcorneal layer, with some superficial acantholysis, which supported the diagnosis of PF (Figure 3). In addition, direct immunofluorescence (DIF) of the perilesional skin yielded intercellular immunoglobulin (Ig) G and C3 deposits in the superficial epidermis (Figure 4). Hematological and biochemical test results and coagulation profiles were all within normal ranges. Furthermore, tests for antinuclear antibodies (ANA) and anti-dsDNA were negative. C3 and C4 levels were not reduced. The Dsg1 and Dsg3 tests were not done.

Prednisolone (15 mg/day) treatment was initiated but was increased to 20 mg/day after 2 weeks because of the slow healing of established lesions. The new dose resulted in only partial improvement. Therefore, we intended to add dapsone because of its corticosteroid-sparing effect. Unfortunately, the patient's glucose-6-phosphate dehydrogenase (G6PD) level was low, and we decided to maintain the previous prednisolone dosage. After 4 weeks, this treatment was effective in reducing desquamation, redness, and healing of the old erosions (Figures S1 and S2). The prednisolone dosage of 20 mg/day lasted for more than 3 weeks before being tapered by 2.5 mg every 2 weeks to eventually reach 5 mg/day. From the third month onward, all skin lesions healed (Figures S3 and S4), and no new blisters or erythema was observed. At the eighth month of the follow-up, the administration of systemic corticosteroids had been discontinued for 1 month and the patient had achieved complete remission.

## 3. Discussion

In contrast to endemic PF, prevalent among inhabitants of the river valleys of rural Brazil that mostly affects children and young adults, sporadic PF rarely develops in childhood. Among the nonendemic pediatric cases of pemphigus, PF seems to be rarer than PV [1]. Less than 40 pediatric PF cases, confirmed by immunofluorescent tests, have been presented in the literature. Some of these patients were neonates, all of whom experienced a transient course of PF over the first few days of life, followed by spontaneous recovery [5, 6]. Placental transfer of maternal antidesmoglein-1 antibodies may play a causative role in neonatal acantholysis. Gradual elimination of these maternal autoantibodies from the neonatal blood circulation promotes the natural remission of pemphigus foliaceus in these newborns. Therefore, neonatal PF should be considered a phenomenon of passive immunity instead of true childhood PF. To better describe the nature of pediatric PF, we recommend further categorizing the condition into the following three subtypes: (1) newborn PF, the cause of which is related to the passive maternal transmission of maternal antibodies; (2) childhood PF (patients aged ≤12 years); and (3) juvenile PF (patients aged 13–18 years).

We conducted a comprehensive literature search of the PubMed database. The keyword “pemphigus foliaceus” was used in combination with “pediatric,” “children,” “child,” “childhood,” “neonate,” “infant,” “juvenile,” or “adolescent.” Endemic patients were excluded from this study. Our search yielded 31 related articles with a total of 32 pediatric PF patients, three of which did not have a full text and were excluded from this review. The 29 remaining cases included four newborns, sixteen child patients, and nine juvenile patients. Excluding the neonatal cases, the youngest PF patient was an 18-months-old girl [7]. Pediatric PF was equally prevalent in both boys and girls (Table 1).

The median time between the onset of skin lesions and definitive diagnosis was 3 months, ranging from a few days to 5 years. The patients were frequently misdiagnosed with impetigo [8], tinea [3, 21], eczema [3], or psoriasis [1, 21], and treatment with neither empirical treatment antibiotics nor antifungal agents proved effective. The cutaneous manifestations of PF in children are similar to those in adults, with fragile, flaccid blisters that quickly rupture into superficial erosions and form crusting plaques. Annular (polycyclic) [3], follicular [27], and erythrodermic [19, 24] variants have been reported in pediatric-onset disease. Mucous membranes are rarely involved in PF, except for mild conjunctivitis in one case [25]. Interestingly, photo-induced PF has also been described in the literature, with four out of 29 patients reporting exacerbation of skin lesions after exposure to sunlight [2, 4, 8, 27].

Previous studies have shown that PF is associated with myasthenia gravis and thymoma in adults. Although this connection was not observed in pediatric patients, concurrent occurrences of PF and Graves' disease have been reported in two juvenile cases [10, 26]. In addition, other autoimmune diseases including dermatomyositis [18] and neuromyelitis optica [22] have also been reported to be possible comorbidities. PF is an autoimmune condition, and the relationship between PF and the potentially higher prevalence of other autoimmune diseases requires further research.

Subcorneal blisters containing acantholytic cells in the blister cavity are pathognomonic for PF. However, the histological features of pediatric PF are sometimes indistinguishable from those of Hailey-Hailey disease [7], psoriasiform dermatitis [4], or toxic epidermal necrolysis [19]. Therefore, DIF remains the gold standard for diagnosis. The DIF test of perilesional skin in the 29 reviewed cases detected IgG, with or without C3, in the intercellular space of the superficial epidermis. Target antigens were searched in 10 of the 29 reviewed cases. Desmoglein-1 was the principal but not the only target. Geller et al. have reported a unique PF case where intercellular IgG deposits attacked desmocollin-1 [2]. Unfortunately, neither immunoblots nor ELISA results for these 10 cases were available for further analysis.

Corticosteroids remain the most effective first-line treatment of PF in current guidelines. As mentioned above, PF in newborns generally resolves without specific treatment. Although localized PF can be successfully treated with topical and/or sublesional injections of corticosteroids, generalized PF requires systemic corticosteroids to induce a clinical response. The optimal corticosteroid dose in pediatric patients with PF has not been established, but prednisone (0.5–2 mg/kg/day), or its equivalents, has proven to be effective in the majority of cases. In our patient, prednisone at a dosage of 1 mg/kg/day showed a slow clinical response. Dapsone was the most commonly prescribed adjuvant therapy in pediatric patients (five cases) but was sometimes ineffective [12]. Other corticosteroid-sparing agents included azathioprine, mycophenolate mofetil, hydroxychloroquine, niacinamide, intravenous (IV) IG, methotrexate, cyclophosphamide, and minocycline. Treatment failure with a combination of systemic corticosteroids and other adjuvant therapies was observed in two children, whose condition then dramatically improved with rituximab infusion. Rituximab, a monoclonal antibody against CD20-positive B cells, is the first FDA-approved biologic agent for the treatment of pemphigus vulgaris in adult patients. Recently, rituximab has proved its safety and efficacy in childhood/juvenile pemphigus patients (both PF and PV) and can be used as first-line therapy in this group [28].

Pediatric PF generally follows a chronic yet benign trajectory. No deaths have been reported in children with PF, and the lesions heal without scarring. Nine children remained free of lesions and required no further treatment for 2 months to more than 3 years. Unfortunately, the others were unable to discontinue systemic drugs, and the majority needed 5–20 mg of prednisone on alternate days. Future case reports are warranted to gain a better understanding of the effective treatment and long-term prognosis for PF in pediatric patients.

## Figures and Tables

**Table 1:** Characteristics of pediatric pemphigus foliaceus patients.

No	Author (year)	Age of onset/sex	Clinical features	Histopathology	DIF	Treatment and outcome
1	Schroeter et al. [8] (1969)	41 mos/M	Crusting plaques on the scalp, neck, back; generalized flaccid blisters on erythematous base sparing only lower extremities	Acantholytic cells lying within a vesicle high in the epidermis	Intercellular staining of the epidermis with IgG	Prednisone started at 140 mg/d + triamcinolone cream wet dressing ⟶ effective control. Discontinued prednisone after approximately 6 months

2	Kahn and Lewis [7] (1971)	18 mos/F	Vesicular, crusting, arcuate, and circinate lesions spread over the trunk, upper arms, thighs, and perineum into the entire body	Subcorneal acantholysis of the granular layer	ND	High potency TCS applied without occlusion 6 times/day for 6 months ⟶ favorable response

3	Sotiriou et al. [9] (1980)	4 yrs/M	Tense bullae on the lower extremities and lower part of the abdomen	Intraepidermal bullae with a few acantholytic cells and chronic inflammation	IgG, C3, and fibrin in the intercellular epidermis with IgM around some blood vessels	Prednisone started at 25 mg/d ⟶ lesions resolved within 2 weeks

4	Levine et al. [10] (1982)	14 yrs/F	Scaly, erythematous, exudative plaques on face, trunk, and legs	Separation of the stratum corneum from the malpighian stratum and acantholysis of the granular cells	Deposition of IgG and C3 in the epidermal intercellular space	Prednisone started at 240 mg/d and tapered to 10 mg/d with an asymptomatic course

5	Jones et al. [11] (1986)	4 yrs/M	Extensive crusting on face and scalp; generalized, papular, scaly eruption on trunk and extremities	Intraepidermal blister at the level of the granular layer, with a few acantholytic cells	Intercellular staining of the high epidermis with IgG	Topical fluorinated steroid ⟶ resolution for 4 months. The relapse required systemic steroids 40 mg/d with gradual taper

6	Yorav et al. [12] (1989)	8 yrs/F	Widespread flaccid vesicles and bullae with superficial erosions on the trunk, shins, and lower legs	Midepidermal cleft containing acantholytic cells	Deposition of IgG and C3 in the epidermal intercellular space	Dapsone 100 mg/d for a month without any clinical improvement. Prednisone 40 mg/d for 3 weeks showed gradual improvement

7	Goodyear et al. [13] (1991)	12 yrs/F	Crusted lesions on the lower limbs; blisters in a linear configuration on the arms, legs and lower abdomen	Separation in the upper epidermis	Intercellular staining of the epidermis with IgG and C3	Prednisolone 80 mg/day showed rapid resolution, which gradually reduced. Azathioprine and sulphapyridine were added as steroid-sparing agents

8	Walker et al. [5] (1995)	Neonatal/M	Denuded skin on the extremities; intact blisters on the right upper arms	Intraepithelial vesicles with eosinophilic infiltrates	Intercellular IgG and C3 deposits in the superficial epidermis	Polysporin ointment twice a day ⟶ completely healed at the sixth week

9	Rosella et al. [14] (1996)	10 yrs/M	Erosions and crusted lesions on the trunk, pubis, ears, and scalp	Subcorneal bulla containing acantholytic epidermal cells. The lower epidermis showed spongiosis and focal acanthosis	Intercellular IgG, IgM, and C3 in the epidermis	Deflazacort 45 mg on alternate days ⟶ partial clinical remission

10	Galambrun et al. [15] (1997)	8yrs/M	Small crusted lesions with raised edges and blisters on the trunk, face, and limbs	Splitting of the upper epidermis, filled with acantholytic cells	IgG and C3 deposits in the upper half of the epidermis	Topical betamethasone ⟶ not responsive. Marked improvement with prednisolone 2 mg/kg/d, but relapse occurred when steroid tapered. Dapsone added ⟶ complete remission in 4 weeks

11	Qureshi et al. [16] (1997)	16 yrs/M	Superficial flaccid bullae and erosions on the upper chest, upper back, and neck	Intraepidermal separation in the upper malpighian layers with acantholytic cells	Intercellular IgG and C3 in the upper stratum Malpighi epidermis	TCS cream + triamcinolone acetonide injection ⟶ well responsive

12	Mehravaran et al. [1] (1998)	7 yrs/F	Generalized erythema, with superficial erosions on the face, trunk, and extremities; intact superficial blisters on the extremities	Subcorneal blister with acantholytic cells without any inflammatory cells in the cleft	Intercellular staining for IgG, IgA, and C3 in the epidermis	Prednisone 50 mg/d showed slow remission. A prednisone and dapsone combination resulted in complete recovery after a month

13	Metry et al. [4] (2002)	3 yrs/M	Serpiginous, crusted, erythematous plaques on face and neck	Subcorneal pustule with upper epidermal acantholysis	Intercellular IgG and C3 in the epidermis	Prednisolone 2 mg/kg/d + HCQ 5 mg/kg/d ⟶ no improvement. Prednisolone 2 mg/kg/d + HCQ 7 mg/kg/d ⟶ well responsive

14	Avalos-Díaz et al. [6] (2000)	Neonatal/M	Erythematous eruption on the trunk and scattered vesicles on his trunk, arms, and legs	Subcorneal vesicles	Intercellular IgG and C3 in the epidermis	Spontaneous resolution within two weeks

15	Hirsch et al. [17] (2003)	Neonatal/F	Erosions on face, ears, chest, and extremities	Cleft within the superficial epidermis with a sparse neutrophilic infiltrate	Intercellular IgG and C3 in the epidermis	Mupirocin 2% + hydrocortisone valerate ⟶ well responsive in 2 days and no additional lesion development

16	Narbutt et al. [18] (2003)	11 yrs/M	Disseminated bullae, vesicles in an annular pattern, and erosions on the extremities, abdomen	Acantholysis and intraepidermal, subcorneal blistering	Intercellular IgG in the epidermis	Cyclophosphamide 1 mg/kg/d + methylprednisolone 1.5 mg/kg/d + HCQ 100 mg/d ⟶ clinical improvement within 4 weeks

17	Connelly et al. [19] (2007)	21 mos/F	Erythroderma; erosions on hands and feet but no evident vesicles/bullae	Subcorneal blister with few acantholytic cells	Granular/linear IgG and C3 on the keratinocyte epidermal surfaces	Solumedrol 2 mg/kg/d ⟶ well responsive but prednisone dose could not be weaned. Rituximab ⟶ dramatic improvement after the second infusion, prednisone was weaned to 0.5 mg/kg/every other day

18	Mlynek et al. [20] (2009)	14 yrs/F	Sharply demarcated crusted erosions and discrete flaccid vesicles on upper trunk, face, and scalp; alopecia	Subcorneal acantholytic clefts within the epidermis	Intercellular IgG in the epidermis	Prednisolone 1 mg/kg/d + IVIg ⟶ well responsive

19	Fariba et al. [21] (2012)	12 yrs/M	Erythroderma with scaling and exudation; mild palmoplantar keratoderma and scales covered the scalp	Subcorneal cleft in the granular layer	Intercellular IgG and C3 in the upper epidermis	Prednisolone 30 mg/d + azathioprine 50 mg/d ⟶ poor response Prednisolone was increased to 50 mg/d ⟶ improvement

20	Salazar et al. [22] (2012)	16 yrs/F	Hyperpigmented patches with hemorrhagic crusting and scale, scattered vesicles, and pustules on the trunk	Subcorneal blister with superficial acantholysis	Intercellular IgG in the epidermis	Prednisone was transitioned to minocycline + nicotinamide

21	Lorente Lavirgen et al. [23] (2012)	Neonatal/F	Flaccid bullae and denuded skin areas on the extremities	ND	ND	Prednisolone 0.5 mg/kg/d for a week + TCS ⟶ completely clear of skin lesions

22	García-Meléndez et al. [24] (2013)	11 yrs/F	Tense blisters on the face progressing slowly to erythroderma with yellow-greenish crusts	Subcorneal bulla with upper epidermal acantholysis	Deposition of C3 and IgG in the stratum spinosum with a beehive pattern	Prednisone 1 mg/kg/d + dapsone 50 mg/d ⟶ satisfactory response

23	Adah et al. [25] (2014)	13 yrs/F	Diffuse, tender, erythematous, desquamating rash on the trunk, face, and extremities; intact flaccid blisters; mild conjunctivitis	Subcorneal blister filled with acantholytic cells and scattered neutrophils	Intercellular IgG, C3, and fibrinogen in the epidermis	Pulse steroids in 3 days (methylprednisolone 500 mg/d) ⟶ rapid improvement. Maintained with prednisone 40 mg/d + MMF 30 mg/kg/d

24	Geller et al. [2] (2016)	11 yrs/F	Erythematous plaques with yellow-brown crusts on the face, upper trunk, and arms	Subcorneal bulla with acantholytic keratinocytes at the base of the blister	Intercellular IgG in upper the epidermis; fine granular linear IgM deposits along the dermoepidermal junction	Prednisone 1 mg/kg/d ⟶ well responsive

25	Laarman and Horii [26] (2017)	15 yrs/F	Flaccid vesicles on the inner upper arms and axillae; superficial erosions with crust on the buttocks, back, chest, face, and scalp	Subcorneal split with eosinophilic spongiosis	Intercellular IgG and C3 in the epidermis, with deposition of C3 along the basement membrane	Prednisone + MMF ⟶ well responsive

26	Loh and Paravar [27] (2017)	17 yrs/M	Follicular pustules on over the body; hyperpigmented, grey, and violaceous crusted papules overlying pink patches on hands, trunk, bilateral cheeks, and nose, sparing the nasolabial folds	Epidermal acanthosis and multifocal areas of eosinophilic spongiosis within the epidermis. Lymphocytic infiltrate with occasional eosinophils in the superficial dermis	Intercellular IgG and C3 in the epidermis	Prednisone 80 mg/d + MMF 2 g/d ⟶ partially responsive. Rituximab added ⟶ significant improvement

27	Evans et al. [3] (2019)	8 yrs/M	Erythematous annular and polycyclic plaques with central clearing and peripheral scaling on the face, trunk, and proximal extremities	Subcorneal vesicle with acantholytic keratinocytes; a few neutrophils in the areas of parakeratosis	Intercellular IgG in the epidermis	Systemic steroids + rituximab ⟶ well responsive

28	Kianfar et al. [28] (2022)	14 yrs/F 16 yrs/M	Not mentioned	Not mentioned	Not mentioned	Systemic steroids + rituximab ⟶ dramatically responsive

29	Our case	42 mos/F	Generalized scaling and crusted erythematous patches; flaccid blisters easily ruptured into diffuse crusted erosions on trunk and extremities	Splits at the level of the subcorneal layer with superficial acantholysis	Intercellular staining of the superficial epidermis with IgG and C3	Prednisolone 20 mg/d ⟶ partial improvement

PF, pemphigus foliaceus; DIF, direct immunofluorescence; mos, months; Ig, immunoglobulin; yrs, years; ND, not done; HCQ, hydroxychloroquine; IV, intravenous; MMF, mycophenolate mofetil.

## Data Availability

The data used to support the findings of this study are included within the article.

## Supplementary Materials

Supplementary Materials Figure S1: partial outcome after 4-week treatment of a pemphigus foliaceus patient with prednisolone 20 mg/day (on the face). Figure S2: partial outcome after 4-week treatment of a pemphigus foliaceus patient with prednisolone 20 mg/day (on the trunk and extremities). Figure S3: the pemphigus foliaceus patient was completely free of skin lesions and did not develop any new lesions (on the face) during the prednisolone tapering period. Figure S4: this pemphigus foliaceus patient was completely free of skin lesions (on the trunk and extremities) and did not develop any new lesions during the prednisolone tapering period.
