# Supplementary material for: Sporadic Pemphigus Foliaceus in a 3-Year-Old Vietnamese Girl: A Case Report and Literature Review
Source: Case Rep Dermatol Med. 2024 Feb 16;2024:6748340. doi: 10.1155/2024/6748340 (PMC10890903; doi:10.1155/2024/6748340)
Supplement: Supplementary Materials — Figure S1: partial outcome after 4-week treatment of a pemphigus foliaceus patient with prednisolone 20 mg/day (on the face). Figure S2: partial outcome after 4-week treatment of a pemphigus foliaceus patient with prednisolone 20 mg/day (on the trunk and extremities). Figure S3: the pemphigus foliaceus patient was completely free of skin lesions and did not develop any new lesions (on the face) during the prednisolone tapering period. Figure S4: this pemphigus foliaceus patient was completely free of skin lesions (on the trunk and extremities) and did not develop any new lesions during the prednisolone tapering period. [file 6748340.f1.zip › Consent form PF pediatric case.pdf]

Patient Consent Form

To record a patient's consent to publication of information relating to them or a relative, in a Wiley publication.

Name of patient: Nhien Ngoc An Huynh

Title of publication/product: Sporadic Pemphigus Foliaceus in a 3-Year-Old Vietnamese Girl: A Case Report and Literature Review

Principal author/editor: Hao Trong Nguyen

Principal author/editor's address: Ho Chi Minh City Hospital of Dermato-Venereology, 2 Nguyen Thong, Ward Vo Thi Sau, District 3, Ho Chi Minh City, Vietnam

I, [Thi Dieu Ngo NAME OF PATIENT / PARENT / GUARDIAN / RELATIVE***] (the "Licensor"), give my permission to use clinical information/video/photographic material relating to [my daughter Nhien ngoc An huynh NAME AND RELATIONSHIP***] in the publication identified above to be published by John Wiley & Sons, Inc. or one of its affiliated companies ("Wiley"), such permission to extend to publication of the information by Wiley and its licensees in all media and languages throughout the world.

***In cases where the patient has died or is incapable of giving consent, consent may be given by the next of kin. If the patient is under the age of 16, consent should be given by a parent or guardian.

I understand that:

The information/video/photographic material will be used only in educational publications intended for health professionals

(1) My name will not be published and Wiley will endeavour to ensure that I cannot be identified from the clinical information, other than in relation to identifiable material (such as videos/photographic material) for which I give consent. However I also understand that there is a low possibility that I may be identified from the clinical information.

(2) If the publication or product is published on an open access basis, I understand that it may be accessed freely throughout the world.

This Agreement shall be governed by, and construed in accordance with: 1) the laws of England and Wales, if the Licensor is located outside of the United States, or 2) the laws of the State of New York, if the Licensor is located in the United States. In relation to any legal action or proceedings to enforce this Agreement or arising out of or in connection with this Agreement each of the parties irrevocably submits to the non-exclusive jurisdiction of the courts: 1) in England and Wales, if the Licensor is located outside of the United States, or 2) in New York, New York, if the Licensor is located in the United States.

***SIGNATURE OF PATIENT/PARENT//GUARDIAN/NEXT OF KIN .... Thi Dieu Ngô [illegible]

***IF PARENT/GUARDIAN/NEXT OF KIN, STATE RELATIONSHIP TO PATIENT.... mother

[ADDRESS] No 18/1/19 Street 1, Ward
Tan Tao A, Binh Tan district HCM

[DATE] 17 Feb 2022

SIGNATURE OF HEALTH PROFESSIONAL OBTAINING PERMISSION (IF APPROPRIATE)

....VO THI DOAN PHUONG

[ADDRESS] Ho Chi Minh City Hospital of Dermato - Venereology
2 Nguyen Thong, Vo Thi Sau Ward,
District 3, Ho Chi Minh, Viet Nam

[DATE] 17 Feb 2022

Note to principal author: The original signed consent form should be retained by the principal author.

Note to health professional: In addition to the consent form, please ensure that any other necessary permissions are cleared for use of the information, including any permissions required for use of information contained in medical records.

MẪU ĐỒNG THUẬN CỦA BỆNH NHÂN

Đây là biểu mẫu ghi lại sự đồng thuận của bệnh nhân về những thông tin có liên quan đến họ hoặc người thân của họ trong ấn phẩm của Wiley.

Tên bệnh nhân: Huỳnh Ngọc An Nhiên

Tựa đề của ấn phẩm/sản phẩm: Sporadic Pemphigus Foliaceus in a 3-Year-Old Vietnamese Girl: A Case Report and Literature Review

Nghiên cứu viên chính/biên tập viên: Nguyễn Trọng Hào

Địa chỉ của nghiên cứu viên chính/biên tập viên: Bệnh viện Da Liễu Thành Phố Hồ Chí Minh. Số 2 Nguyễn Thông, phường Võ Thị Sáu, Quận 3, Thành phố Hồ Chí Minh

Tôi tên là ....Ngô Thị Diệu.. Tôi đồng thuận cho phép sử dụng thông tin lâm sàng/đoạn phim/hình ảnh có liên quan đến ...Con...của...tôi........ (mối quan hệ) là ..Huỳnh...Ngọc An Nhiên...(tên) trong ấn phẩm trên để được xuất bản bởi công ty John Wiley & Sons hoặc các công ty có liên quan (như "Wiley"). Sự cho phép của Tôi bao gồm cả quyền xuất bản thông tin bởi Wiley và những chủ thể được cấp bản quyền của Wiley trên các phương tiện truyền thông và ngôn ngữ trên thế giới.

*** Trường hợp bệnh nhân đã mất, hoặc không thể có khả năng đưa ra đồng thuận, thì người có quan hệ gần nhất có thể đồng thuận thay. Nếu bệnh nhân dưới 16 tuổi, cha mẹ hoặc người giám hộ có thể đồng thuận thay.

Tôi hiểu rằng:

Các thông tin/đoạn phim/hình ảnh chỉ được dùng trong những ấn phẩm mang tính giáo dục cho nhân viên y tế.

(1) Tên của Tôi không được nêu ra và Wiley sẽ cố hết sức để chắc chắn Tôi không thể được định danh từ các thông tin lâm sàng cũng như các thông tin có thể định danh khác (như đoạn phim hay hình ảnh) trong các ấn phẩm mà Tôi đồng thuận. Tuy nhiên, Tôi cũng hiểu rằng có khả năng, dù là thấp, Tôi có thể được xác định từ các thông tin lâm sàng.

(2) Nếu ấn phẩm hoặc sản phẩm được xuất bản trên nền tảng mở, Tôi hiểu rằng nó có thể được truy cập tùy thích ở bất cứ nơi nào trên thế giới.

Đồng thuận này tuân thủ theo Luật pháp của Anh Quốc và Xứ Wales, nếu cơ quan cấp phép nằm ngoài Hợp Chúng Quốc Hoa Kỳ, hoặc theo Luật Pháp của Bang New

York, nếu cơ quan cấp phép nằm bên trong Hợp Chúng Quốc Hoa Kỳ. Trường hợp có liên quan đến pháp lý hoặc tố tục khi thực hiện bản đồng thuận này hoặc các phát sinh hoặc liên kết với bản đồng thuận này, các bên liên quan không thể hủy bỏ quyền phán xét không hạn chế của Tòa án của Anh Quốc và Xứ Wales, hoặc Tòa án New York.

*** CHỮ KÝ CỦA BỆNH NHÂN/CHA MẸ/NGƯỜI GIÁM HỘ/NGƯỜI CÓ QUAN HỆ GẦN NHẤT ...... Ngô Thị Diệu ......

*** NẾU LÀ CHA MẸ/NGƯỜI GIÁM HỘ/NGƯỜI CÓ QUAN HỆ GẦN NHẤT, THÌ NÊU RÕ QUAN HỆ VỚI BỆNH NHÂN ...... Mẹ ruột ......

ĐỊA CHỈ 18/1/19 đ số 1 Tân Tạo A Bình Tân

____

____

NGÀY 17/2/2022

CHỮ KÝ CỦA NHÂN VIÊN Y TẾ THU THẬP ĐỒNG THUẬN (NẾU PHÙ HỢP)

...... Võ Thị Đoan Phượng ......

ĐỊA CHỈ Bệnh viện Da Liễu Thành phố Hồ Chí Minh

Số 2 Nguyễn Thông - F. Võ Thị Sáu Q3 TP Hồ Chí Minh

____

NGÀY 17/2/2022

Nghiên cứu viên chính lưu ý: bản chính của bản đồng thuận đã có chữ ký cần được lưu giữ bởi nghiên cứu viên chính.

Các nhân viên y tế lưu ý: ngoài bản đồng thuận, nhân viên y tế hãy chắc chắn có sự cho phép khi sử dụng những thông tin, bao gồm cả sự cho phép sử dụng thông tin trong hồ sơ ghi chép y khoa.

Bản đồng thuận này là bản dịch tiếng Việt, do nhóm tác giả ấn phẩm thực hiện dựa trên Bản đồng thuận gốc bằng tiếng Anh. Nhóm tác giả ấn phẩm đảm bảo tính trung thực trong quá trình dịch sang tiếng Việt của Bản đồng thuận gốc. Người ký bản đồng thuận này cũng đồng ý ký bản đồng thuận gốc bằng tiếng Anh theo hướng dẫn của nhóm tác giả ấn phẩm./
