# Supplementary material for: Sporadic Pemphigus Foliaceus in a 3-Year-Old Vietnamese Girl: A Case Report and Literature Review
Source: Case Rep Dermatol Med. 2024 Feb 16;2024:6748340. doi: 10.1155/2024/6748340 (PMC10890903; doi:10.1155/2024/6748340)
Supplement: Supplementary Materials — Figure S1: partial outcome after 4-week treatment of a pemphigus foliaceus patient with prednisolone 20 mg/day (on the face). Figure S2: partial outcome after 4-week treatment of a pemphigus foliaceus patient with prednisolone 20 mg/day (on the trunk and extremities). Figure S3: the pemphigus foliaceus patient was completely free of skin lesions and did not develop any new lesions (on the face) during the prednisolone tapering period. Figure S4: this pemphigus foliaceus patient was completely free of skin lesions (on the trunk and extremities) and did not develop any new lesions during the prednisolone tapering period. [file 6748340.f1.zip › Figure S4 (2).docx]

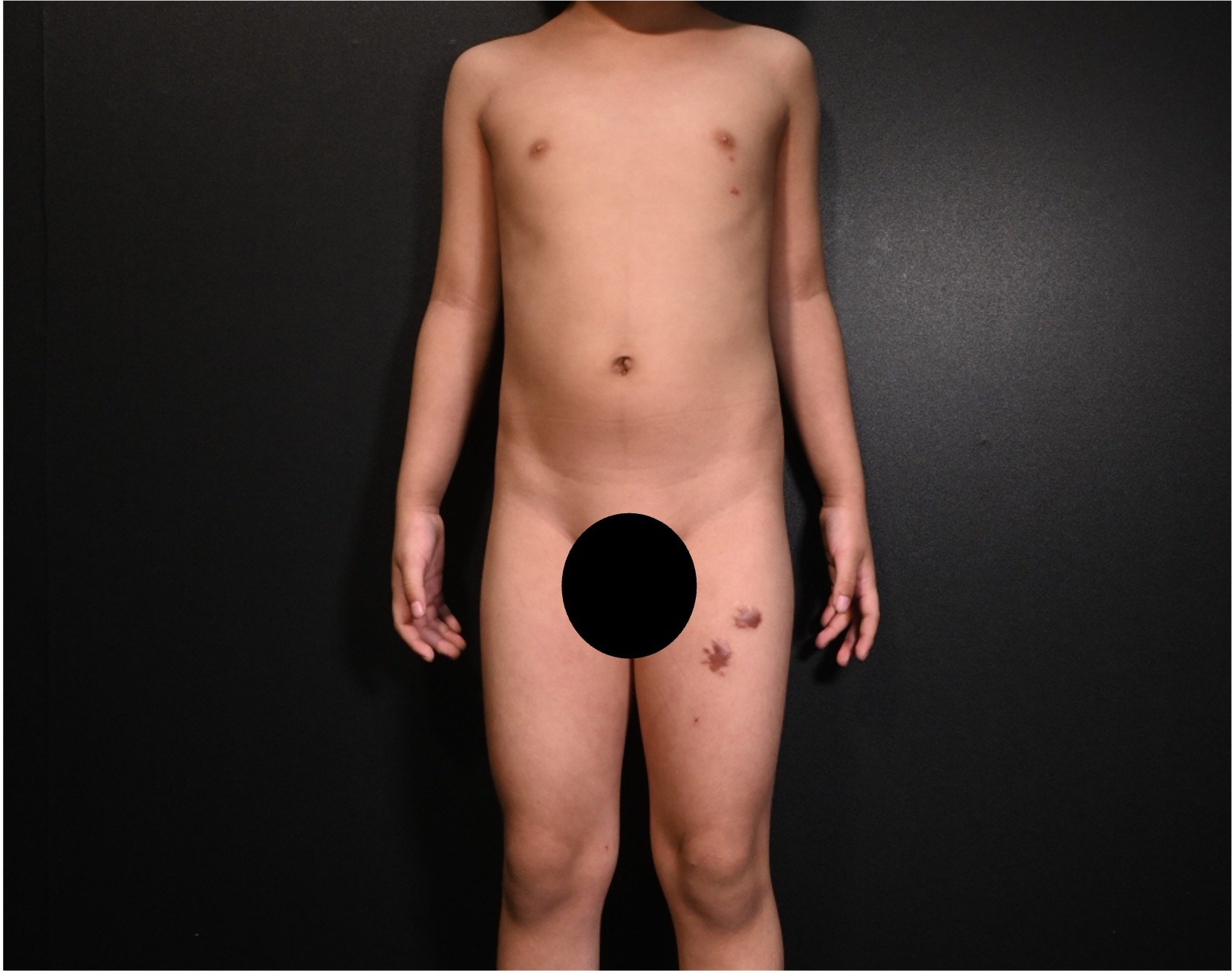


Figure S4. This pemphigus foliaceus patient was completely free of skin lesions (on the trunk and extremities) and did not develop any new lesions during the prednisolone tapering period.
